# Absolute Measurement of the Refractive Index of Water by a Mode-Locked Laser at 518 nm

**DOI:** 10.3390/s18041143

**Published:** 2018-04-09

**Authors:** Zhaopeng Meng, Xiaoyu Zhai, Jianguo Wei, Zhiyang Wang, Hanzhong Wu

**Affiliations:** 1 School of Computer Software, Tianjin University, Tianjin 300072, China; mengzp@tju.edu.cn (Z.M.); 1013202057@tju.edu.cn (X.Z.); jianguo@tju.edu.cn (J.W.); 2 National Ocean Technology Center, Tianjin 300112, China; 3 School of Marine Science and Technology, Tianjin University, Tianjin 300072, China; wzy_sxn@tju.edu.cn

**Keywords:** refractive index of water, frequency comb, interferometry, metrology

## Abstract

In this paper, we demonstrate a method using a frequency comb, which can precisely measure the refractive index of water. We have developed a simple system, in which a Michelson interferometer is placed into a quartz-glass container with a low expansion coefficient, and for which compensation of the thermal expansion of the water container is not required. By scanning a mirror on a moving stage, a pair of cross-correlation patterns can be generated. We can obtain the length information via these cross-correlation patterns, with or without water in the container. The refractive index of water can be measured by the resulting lengths. Long-term experimental results show that our method can measure the refractive index of water with a high degree of accuracy—measurement uncertainty at 10^−5^ level has been achieved, compared with the values calculated by the empirical formula.

## 1. Introduction

During the past decades, marine technology has attracted great attention both as a field of research, and for economical reasons [1]. The resources hidden underwater, such as coal, oil, and natural gas, are receiving increasing interest because of depleting resources on land. Scientists all over the world have developed a wealth of marine instruments, so as to make sailing, as well as resource exploration, convenient and efficient. The acoustic method is the most-widely used technique, owing to a lower attenuation when travelling underwater; however, due to the multipath effect, considerable noise could exist in the signal returns [2,3,4], resulting in a very complicated data process. In addition, the measurement resolution of the acoustic wave is strongly limited by beam size and divergence. The electro-magnetic wave (~GHz) can be exploited in short distance measurements underwater (tens of meters), based on phase measurement and power attenuation [5,6]. In the case of the phase measurement, accuracy and precision cannot be very high on account of the larger electro-magnetic wavelength. In terms of power attenuation, power-based measurements are negatively affected by extreme environments underwater. In short-path underwater measurements, optical waves, with a transmission window of between 480 nm and 540 nm, are an ideal candidate [7,8,9]. With excellent laser alignment and inherent high power, the underwater optical technique has shown itself to be highly effective in marine applications [10]. Different from acoustic and electro-magnetic waves, one basic issue associated with the optical method is the correction of the refractive index of water [11].

Measurement of the refractive index of water is of fundamental importance in the application of optical underwater techniques, and various methods have been proposed to address the issue. Analogous with measurements of the refractive index of air, indirectly, the refractive index of water can be corrected based on a knowledge of the wavelength, temperature, and density of the water, by using an empirical formula [12,13]. Directly, the refractive index of water can be measured using the methods of refraction and interferometrics. Classical schemes mostly rely on Snell’s law, e.g., Abbe refractometer [14], Pulfrich refractometer (based on the measurement of the deflection angle of the incident ray on a V-shaped prism [15]), and numerous other variants [16,17]. For these methods, knowledge of the refractive index of the prism is required, as well as the precise determination of the corresponding angles. In general, the measured angles can be determined by autocollimators; however, the resulting system is very complicated, and often involves moveable elements. Measurement uncertainty is at 10^−4^ level. In contrast to refraction methods, the interferometric technique, based on fringe counting, can provide better resolution and lower measurement uncertainty (better than 10^−5^). Traditionally, a water container (sample cell) is placed in the measurement path of the interferometer [18,19], a Michelson interferometer or Mach-Zehnder interferometer. The refractive index of water can be determined by measuring the optical path difference between the reference and measurement beam, with or without water in the container. Consequently, the geometrical length of the container is required. In this case, the thermal expansion of the water container can make considerably increase measurement uncertainty. To overcome this problem, scientists have proposed many solutions, e.g., the auto-compensating interferometer [20], and the multi-step interferometer [21], etc. B. Richerzhagen presented a configuration of a Michelson interferometer without compensation, where the measurement mirror is fixed in the water [22]. The measurement mirror should be well thermally isolated from the water container, and should be fixed on a moveable stage, to yield an absolute measurement of the refractive index. In addition, dispersive interferometry with a low coherence source [23], and the technique of fiber sensors [24] have also been reported.

Frequency combs have achieved huge success in many applications since their invention [25]. Benefitting from extremely high accuracy and precision of the frequency elements, frequency combs have advanced a wealth of fields, including precision spectroscopy [26], time/frequency transfer [27], and length metrology [28], etc. Length is a fundamental quantity in science and technology. During the past decade, frequency combs have revolutionized techniques of absolute distance measurement, as well as measurements of the quantity related to length, such as thickness [29], vibration/speed [30], surface profiling [31], and refractive index [32], etc. We focus on the measurement of refractive indices in this paper. To date, the measurement of the refractive index of air using a frequency comb has already achieved a 10^−9^ uncertainty [33], far surpassing those obtained using empirical formulas. Thickness and refractive index measurements of solids (glass, wafer) using frequency comb have also been in development for more than a decade [34,35,36], and various methods have been investigated in great depth. However, there are few reports about measurements of the refractive index of liquids using frequency comb.

In this study, we carried out absolute measurements of the refractive index of water using a frequency comb at 518 nm. We develop an interferometric system, where both the measurement and reference mirrors of the Michelson interferometer are under water in a quartz-glass container. Consequently, the expansion compensation of the water container is not required. Cross-correlation patterns can be obtained with a scanning stage, which can be processed to determine the corresponding lengths. The refractive index of water can be measured via the obtained lengths. Experimental results showed an agreement at 10^−5^ level, compared with the empirical formula [13].

## 2. Materials and Methods

**Figure 1:**
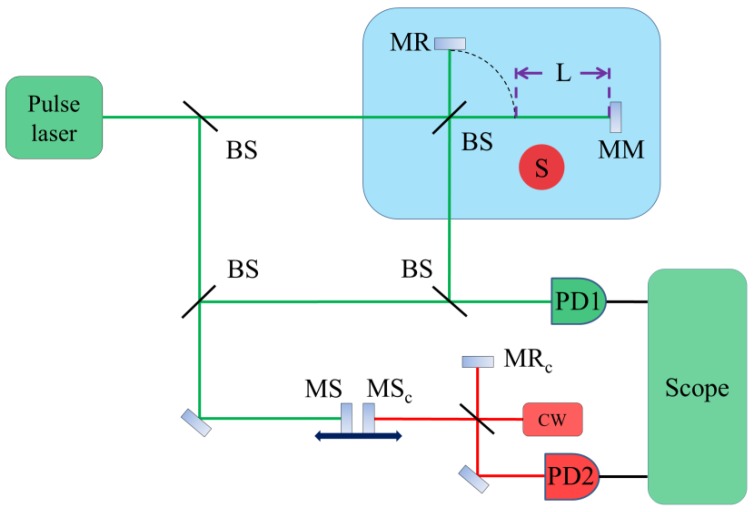
Experimental setup. BS: beam splitter; MR: reference mirror; MM: measurement mirror; S: sensors; PD: photodetector; MS: scanning mirror; MR_c_: reference mirror of the cw interferometer; MS_c_: scanning mirror of the cw interferometer; cw: continuous wave laser.

The experimental setup is shown in Figure 1. The frequency comb (Menlosystem FC1000 (Menlosystem, Martinsried, Germany), 100 MHz repetition frequency, 500 mW maximum output power, 518 nm center wavelength) emits a pulsed laser, which is split at a beam splitter. One part is directed into the Michelson interferometer, which can introduce a measured length L. Please note that the entire Michelson interferometer is placed in the water container, which means that the reference path and the measurement path are thermally expanded at the same time. In addition, the thermal expansion coefficient of quartz glass is relatively lower. Therefore, the system can automatically compensate for the thermal expansion of the water container. The other part is scanned by using a moving stage (PI M521, PI, Karlsruhe, Germany), 200 mm travel range), and is then combined with the output of the Michelson interferometer at a beam splitter. A photo-detector (PD1, Thorlabs APD430A, Thorlabs, NJ, USA) is used to detect the combined beam, and an oscilloscope (LeCroy 640Zi, LeCroy, NYC, USA) is used to measure and store the cross-correlation patterns. To obtain the precise position of the scanning mirror (MS), a fringe counting interferometer based on a cw laser (Thorlabs HRS015B, Thorlabs, NJ, USA), 632.991 nm, 2 MHz frequency stability) is used as the distance meter. The temperature and the density of water are measured by Valeport Mini SVP in real time, which can be used to calculate the refractive index of water with the empirical formula (i.e., Harvey formula) [13]. Please note that the Harvey formula is used in the case of pure water. In our experiments, we used the tap water, which is sufficiently clean for the Harvey formula to be applicable.

We measure the refractive index of water with two steps. First, the water container is not filled with water. We can obtain a length value *L_a_* in air, and *L_a_* = *n_a_L*. *n_a_* is the group refractive index of air, which can be corrected by using empirical formulae, and *L* is the geometrical length difference between the reference and measurement beams of the Michelson interferometer. Second, the water container is full with water. Considering the group refractive index of water, we can obtain a length value *L_gw_*, i.e., *n_gw_L*, where *n_gw_* is the group refractive index of water. In the case of phase refractive index at a certain wavelength, the corresponding length value *L_p_* can be expressed as *n_p_L*, where *n_p_* is the corresponding phase refractive index of water. Consequently, the group refractive index of water *n_gw_* can be calculated as:

(1) $$n_{gw}=\frac{L_{gw}}{L}=\frac{L_{gw}}{L_{a}/n_{a}}$$

For a certain wavelength, the phase refractive index of water can be indicated as:

(2) $$n_{p}=\frac{L_{p}}{L}=\frac{L_{p}}{L_{a}/n_{a}}$$

After we obtain the values of *L_gw_*, *L_p_*, *L_a_* and *n_a_*, the group and phase refractive indices of water can be measured.

## 3. Results

**Figure 2:**
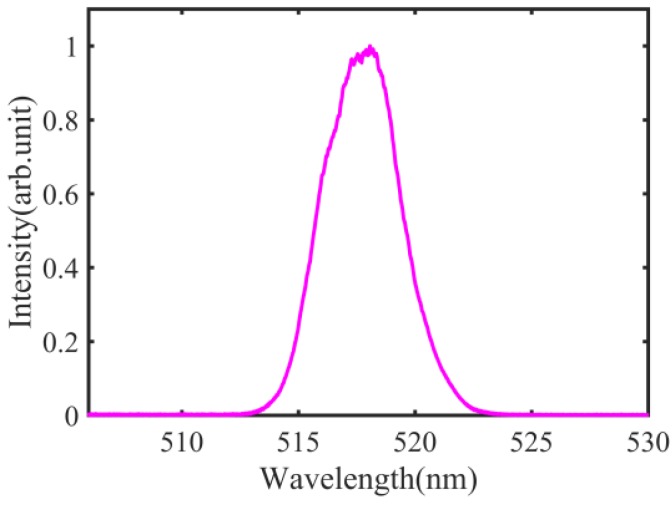
The source spectrum.

The source spectrum is shown in Figure 2, with a center wavelength of approximately 518 nm and a spectral width of around 4 nm. The environmental parameters are: 19.2 °C temperature, 1032.5 hPa pressure, and 35% humidity. The group refractive index of air *n_a_* can be calculated as 1.00029157 by Ciddor formula [37].

### 3.1. Measurement of Group Refractive Index of Water

**Figure 3:**
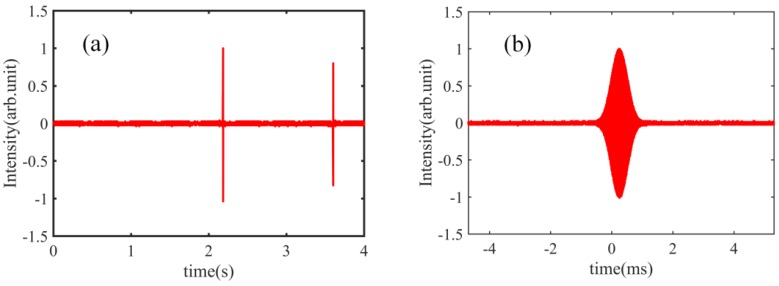
(**a**) Cross-correlation patterns corresponding to the reference mirror and the measurement mirror, without water in the container; (**b**) detailed observation of the cross-correlation pattern, without water in the container.

In this section, we describe the measurement of the group refractive index of water. The speed of the moving stage is set to 50 mm/s, i.e., travel time for a single stroke is 4 s. First, the water container is not filled with water. We can obtain a pair of cross-correlation patterns, shown in Figure 3a, which correspond to the reference and measurement mirrors respectively. Figure 3b shows a detailed observation of the cross-correlation pattern; in it, we see that the pulse width is about 100 μm (333 fs).

**Figure 4:**
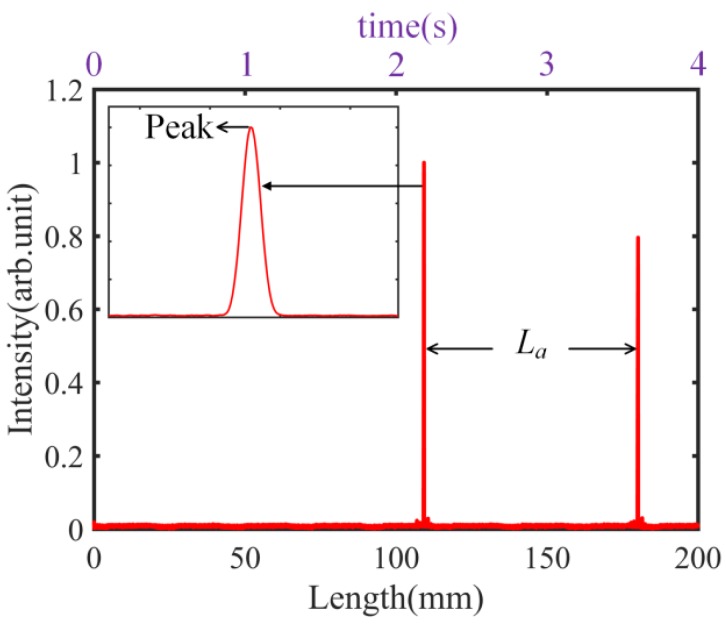
Results of Hilbert transform.

Performing a Hilbert transform of the cross-correlation patterns in Figure 3a, we can obtain the results shown in Figure 4, where the inset is the curve expansion. Based on the peak positions, the length *L_a_* can be measured, using a fringe counting interferometer, at 70.783 mm. Therefore, length *L* can be calculated as 70.783 mm/1.00029157, i.e., 70.762 mm.

**Figure 5:**
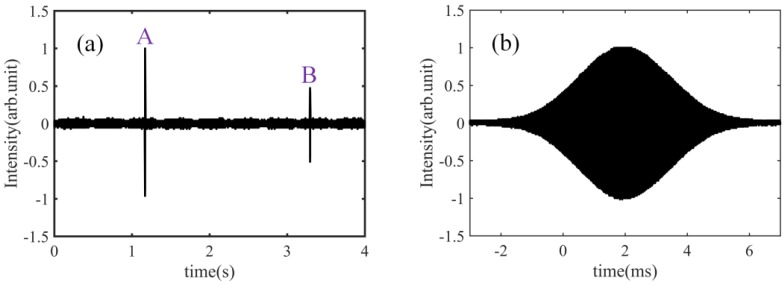
(**a**) Cross-correlation patterns corresponding to the reference mirror and the measurement mirror, with water in the container; (**b**) detailed observation of the cross-correlation pattern, with water in the container.

Second, the container is full with water. The obtained cross-correlation patterns are indicated in Figure 5a. Figure 5b is the expansion of a single cross-correlation pattern in the time axis. We find that, the cross-correlation pattern is obviously broadened to about 350 μm (1.2 ps). This broadening is due to water dispersion. The length *L_gw_* can be measured by the same process as that of Figure 4, and is 96.676 mm. Based on Equation (1), the group refractive index of water can be thus calculated as: 96.676/70.762 = 1.36621. The water conditions were: 14.4 °C temperature and 999.2 kg/m^3^ density, and based on the empirical formula, the group refractive index of water can be calculated to be 1.36622. Finally, we find a difference of 1 × 10^−5^ between our result and that obtained using the empirical formula. Please note that the empirical formula in this work refers to Reference [13].

**Figure 6:**
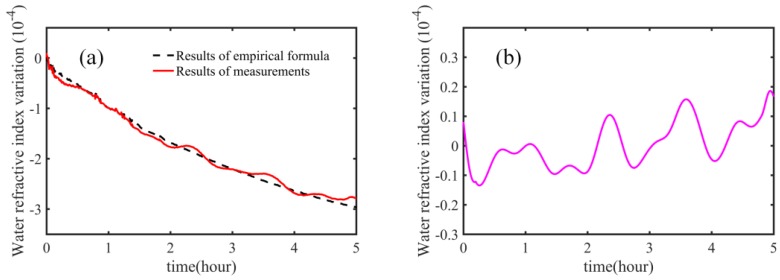
Long time measurements of the group refractive index of water. (**a**) Results obtained using the empirical formula and our measurements; red solid line: result of our method; black dashed line: result of the empirical formula; (**b**) difference between the results of empirical formula and our measurements.

We performed a measurement over a 5-h period; the results are shown in Figure 6. In Figure 6a, the red solid line indicates the results obtained by our method, and the black dashed line represents results obtained using the empirical formula. Please note that, for convenience of display, both results are shifted by −1.36622. We observed that the group refractive index of water changed by up to around 3 × 10^−4^ in a 5-h period. Figure 6b shows the difference between the results obtained using the empirical formula and our method. In long-term experiments, we observed the difference to be be well below 2 × 10^−5^.

### 3.2. Measurement of Phase Refractive Index of Water

In this section, we measure the phase refractive index of water at a particular wavelength. Different from the Section 3.1, here we used a Fourier transform to determine the distance for each wavelength. In fact, as a classical method of data processing, Fourier transforms can also be used in Section 3.1. In that case, the length can be measured through the slope of the unwrapped phase, and the results are nearly the same.

**Figure 7:**
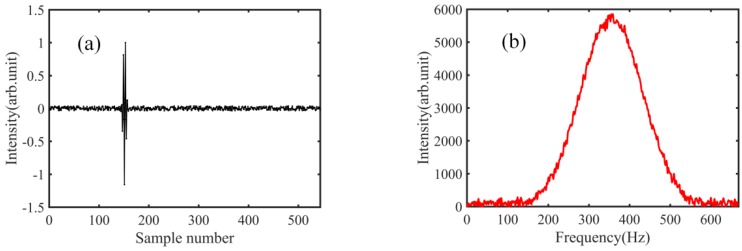

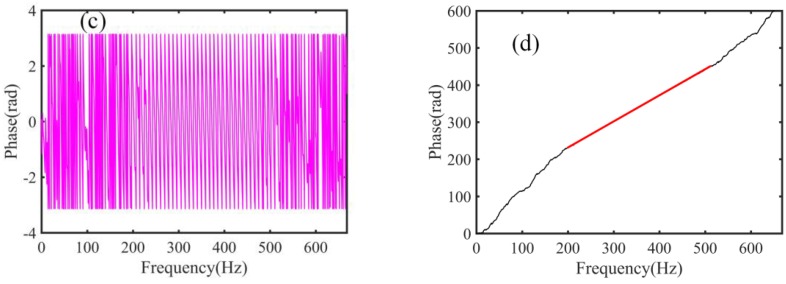
Fourier transform data process. (**a**) Data after the sub sampling; (**b**) frequency spectrum of Fourier transform; (**c**) wrapped phase of Fourier transform; (**d**) unwrapped phase of Fourier transform.

Assuming that the phase of the wavelength *λ* is *φ*_0_ for the reference mirror, and *φ*_1_ for the measurement mirror, the length *L_p_* can be calculated as (*φ*_1_ − *φ*_0_)/(2*π*) × *λ* for the wavelength *λ* [38,39]. Please note that *φ*_1_ and *φ*_0_ are the unwrapped phases. The data process of the Fourier transform is shown in Figure 7. The first cross-correlation pattern, (A) in Figure 5, is picked up, and zero padding is needed. To enhance process efficiency and save memory, we subsample the raw data, which is a mature technique widely used in the signal processing [40,41]; the resulting curve is shown in Figure 7a. Please note that the subsampling coefficient *N*_0_/*N*_1_ should be defined carefully; it must meet the condition that the frequency spectrum of the Fourier transform be completely located within a range from 0 to *f_s_*/2 (without spectrum leakage), analogous to the demonstration mentioned in Reference [42]. *N*_0_ is the sample number of the raw data (3 × 10^6^ in our experiments), *N*_1_ is the sample number after subsampling (544 in our experiments), and *f_s_* is the sampling rate. Figure 7b shows the frequency spectrum, and we find that no spectrum leakage exists. Figure 7c,d shows the wrapped phase and unwrapped phase respectively. The data process of the second cross-correlation pattern (B in Figure 5) is the same as that shown in Figure 7. After the phases are obtained, *L_p_* can be determined, and the phase refractive index can be measured based on Equation (2).

**Figure 8:**
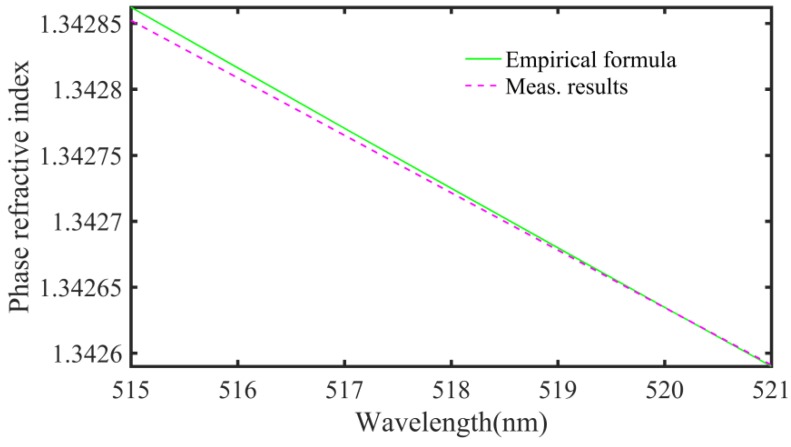
Results of the phase refractive index measurement; pink dashed line: result of our method; green solid line: result of the empirical formula.

With water conditions of 14.4 °C and 999.2 kg/m^3^, the experimental results of phase refractive index measurements in the spectral range from 515 nm to 521 nm are shown in Figure 8. The pink dashed line indicates the results obtained using our method, and the green solid line shows the those from the empirical formula. In the range from 515 nm to 521 nm, the difference between our method and the empirical formula can be less than 1 × 10^−5^. We also find that water dispersion is very significant when green light transmits in water (~2.5 × 10^−4^ in the 6-nm spectral range), and that the pulse can be strongly broadened.

**Figure 9:**
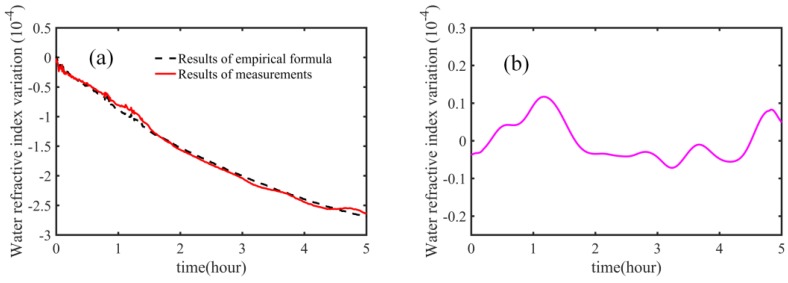
Time measurements of the phase refractive index of water at 518 nm. (**a**) Results of empirical formula and our measurements; red solid line: result of our method; black dashed line: result of the empirical formula; (**b**) difference between the results of empirical formula and our measurements.

We carried out 5-h experiments; the experimental results for 518 nm wavelength (vacuum wavelength) are shown in Figure 9. In Figure 9a, the red solid line indicates the results obtained using our method, and the black dashed line represents those results obtained using the empirical formula. Please note that for convenience of display, both the results are shifted by −1.34272. We found that the results obtained using our method and those from the empirical formula changed under the same law, and the phase refractive index of water at 518 nm varied by up to about 2.5 × 10^−4^ in a 5-h period. Figure 9b shows the difference between the results obtained using the empirical formula and our method. In long-term experiments, we found measurement uncertainty to be well below 1.2 × 10^−5^.

## 4. Uncertainty evaluation

Based on Equation (1), the measurement uncertainty of *n_gw_* is related to *L_gw_*, *L_a_* and *n_a_*, and can be calculated as:

(3) $$u_{n_{gw}}{}^{2}={\left(\frac{L_{gw}}{L_{a}}u_{n_{a}}\right)}^{2}+{\left(\frac{n_{a}}{L_{a}}u_{L_{gw}}\right)}^{2}+{\left(\frac{n_{gw}}{L_{a}}u_{L_{a}}\right)}^{2}$$

The first term of Equation (3) is related to the refractive index of air *n_a_* based on the Ciddor formula. This part is related to the uncertainty of the Ciddor formula itself, the uncertainty of the sensor network, and the stability of the environment. Considering that the total optical path is relatively short, the inhomogeneity of the environment can be neglected. The average value of *L_gw_* is 96.664 mm in 5-h experiments, and *L_a_* equals to 70.783 mm. This part, dependent upon *n_a_*, can thus be evaluated to 1.4 × 10^−8^, which is negligible. The second term of Equation (3) is related to the uncertainty of *L_gw_*, which can be affected by the stability of the water and the algorithm of the data process. We fast performed five measurements; the stability of the measurement of *L_gw_* is 1.3 μm (standard deviation). The second term can be calculated to 1.8 × 10^−5^. The third term of Equation (3) is related to the uncertainty of *L_a_*, and the short-term stability of *L_a_* is 0.6 μm (standard deviation). This part can be estimated to be 1.2 × 10^−5^. The expansion coefficient is 5 × 10^−7^/°C. The temperature change is about 3.5 °C (from 14.4 °C to 17.9 °C) in long-term experiments. Thermal expansion can be calculated at 0.1 μm, corresponding to 1.8 × 10^−6^ uncertainty. Finally, the combined uncertainty can be calculated to be 2.2 × 10^−5^, with a coverage factor of k=1, which shows a good agreement with the results in Figure 6b.

Based on Equation (2), the measurement uncertainty of *n_p_* is related to *L_p_*, *L_a_* and *n_a_*, and can be calculated as:

(4) $$u_{n_{p}}{}^{2}={\left(\frac{L_{p}}{L_{a}}u_{n_{a}}\right)}^{2}+{\left(\frac{n_{a}}{L_{a}}u_{L_{p}}\right)}^{2}+{\left(\frac{n_{p}}{L_{a}}u_{L_{a}}\right)}^{2}$$

As mentioned above, the first term of Equation (4) can be neglected. Considering the second term, the short-term stability of *L_p_* is 0.45 μm, corresponding to 0.64 × 10^−5^ uncertainty of the refractive index. The third term of Equation (4) can be calculated to 1.1 × 10^−5^ uncertainty. The uncertainty due to thermal expansion is also 1.8 × 10^−6^. Therefore, combined uncertainty can be evaluated to be 1.3 × 10^−5^, with a coverage factor of *k* = 1, which shows a good agreement with the results in Figure 9b.

## 5. Conclusions

In this work, we present a method which enables the absolute measurement of the refractive index of water using a frequency comb. Based on changes of the optical path in air and water, the refractive index of water can be determined with high precision, which is of fundamental importance in the optical method when used underwater. Our results show a measurement uncertainty of 10^−5^, that is to say, the corresponding measurement uncertainty is only several mm when we measure a distance of up to 100 m underwater. This kind of performance can satisfy most applications. Thanks to the low power attenuation of blue-green light underwater, frequency-comb instruments show great promise for marine technology, significantly improving upon the current technologies. However, the entire system is relatively expensive on account of the cost of the frequency comb. The development of a portable and low-cost frequency comb would solve this problem [43,44].
